# Engagement, Use, and Impact of Digital Mental Health Resources for Diverse Populations in COVID-19: Community-Partnered Evaluation

**DOI:** 10.2196/42031

**Published:** 2022-12-07

**Authors:** Kenneth Wells, April Denise Thames, Alexander S Young, Lily Zhang, MarySue V Heilemann, Daniela Flores Romero, Adrian Oliva, Felica Jones, Lingqi Tang, Melissa Brymer, Thomas Elliott, Armen Arevian, Together for Wellness/Juntos Collaborators and Writing Group

**Affiliations:** 1 Research Center for Health Services and Society Jane and Terry Semel Institute for Neuroscience and Human Behavior University of California, Los Angeles Los Angeles, CA United States; 2 Department of Psychiatry and Biobehavioral Sciences University of California, Los Angeles Los Angeles, CA United States; 3 Department of Health Policy and Management Fielding School of Public Health University of California, Los Angeles Los Angeles, CA United States; 4 School of Nursing University of California, Los Angeles Los Angeles, CA United States; 5 Healthy African American Families II Los Angeles, CA United States; 6 National Clinician Scholars Program Division of General Internal Medicine and Health Services Research, Department of Medicine University of California, Los Angeles Los Angeles, CA United States; 7 Chorus Innovations, Inc Long Beach, CA United States; 8 See Acknowledgements

**Keywords:** digital mental health, prevention, COVID-19, depression, hotline use, health disparity, community health, public health, health resource, mental well-being, ethnic, website engagement, minority population, digital resource

## Abstract

**Background:**

The COVID-19 pandemic increased disparities for communities burdened by structural barriers such as reduced affordable housing, with mental health consequences. Limited data are available on digital resources for public mental health prevention during the COVID-19 pandemic.

**Objective:**

The study aim was to evaluate engagement in and impact of free digital resources on the Together for Wellness/Juntos por Nuestro Bienestar (T4W/Juntos) website during COVID-19 in California.

**Methods:**

A pilot evaluation of T4W/Juntos was performed, with partner agencies inviting providers, clients, and partners to visit the website and complete surveys at baseline (September 20, 2021, to April 4, 2022) and at 4-6–week follow-up (October 22, 2021, to May 17, 2022). Website use was assessed by three engagement items (ease of use, satisfaction, relevance), comfort in use, and use of six resource categories. Primary outcomes at follow-up were depression and anxiety (scores≥3 on Patient Health Questionnaire-2 item [PHQ2] and Generalized Anxiety Disorder-2 item [GAD2] scales). Secondary outcomes were post-pre differences in PHQ2 and GAD2 scores, and use of behavioral health hotlines and services the month before follow-up.

**Results:**

Of 366 eligible participants, 315 (86.1%) completed baseline and 193 (61.3%) completed follow-up surveys. Of baseline participants, 72.6% identified as female, and 21.3% identified as lesbian, gay, bisexual, transgender, queer/questioning, and others (LGBTQ+). In terms of ethnicity, 44.0% identified as Hispanic, 17.8% as African American, 26.9% as non-Hispanic white, and 11.4% as other ethnicity. Overall, 32.7% had moderate anxiety or depression (GAD2/PHQ2≥3) at baseline. Predictors of baseline website engagement included being Hispanic versus other race/ethnicity (*β*=.27, 95% CI .10-.44; *P*=.002) and number of COVID-19–related behavior changes (*β*=.09, 95% CI .05-.13; *P*<.001). Predictors of comfort using the website were preferring English for website use (odds ratio [OR] 5.57, 95% CI 2.22-13.96; *P*<.001) and COVID-19–related behavior changes (OR 1.37, 95% CI 1.12-1.66; *P*=.002); receiving overnight behavioral health treatment in the prior 6 months (OR 0.15, 95% CI 0.03-0.69, *P*=.015) was associated with less comfort in website use. The main predictor of depression at follow-up (PHQ2≥3) was baseline depression (OR 6.24, 95% CI 2.77-14.09; *P*<.001). Engagement in T4W/Juntos was associated with lower likelihood of depression (OR 0.54, 95% CI 0.34-0.86; *P*=.01). Website use the month before follow-up was associated with a post-pre reduction in PHQ2 score (*β*=–.62, 95% CI –1.04 to –0.20; *P*=.004). The main predictor of GAD2≥3 at follow-up was baseline GAD2≥3 (OR 13.65, 95% CI 6.06-30.72; *P*<.001). Greater baseline website engagement predicted reduced hotline use (OR 0.36, 95% CI 0.18-0.71; *P*=.004).

**Conclusions:**

Ethnicity/language and COVID-19–related behavior changes were associated with website engagement; engagement and use predicted reduced follow-up depression and behavioral hotline use. Findings are based on participants recommended by community agencies with moderate follow-up rates; however, significance was similar when weighting for nonresponse. This study may inform research and policy on digital mental health prevention resources.

## Introduction

### Background

The COVID-19 pandemic increased disparities for communities burdened by structural barriers such as shortage of affordable housing or work opportunities, with mental health consequences [1]. Stressors related to COVID-19 include loss/grief, self-quarantining, physical distancing, social isolation, business and school closures, financial impacts, lack of access to basic needs, and stress, including that related to racial discrimination, with mental health consequences [2]. There is also documentation of provider stress such as burnout, fear of infection, and loss [3]. There are racial/ethnic disparities in prevalence and economic impacts, and disparities exist for other marginalized groups such as lesbian, gay, bisexual, transgender, queer/questioning and others (LGBTQ+), rural farm workers, and persons with mental health challenges [1,3-6]. These impacts are noted across age groups, with concerns about school/work-related distress, substance use, suicidality, abuse/violence, and overall mental health consequences [7,8], highlighting the importance of public-facing interventions.

With advances in social media and digital technology, the importance of digital interventions for public health is clear; some countries use digital resources to promote mental health prevention, including in youth portals [9]. Such strategies may reduce the need for services, while increasing access to services for those in need [10]. There are limited data on the effectiveness of digital interventions for public mental health and in disasters [10]. Over the past decade, mental health care has been supplemented by mobile mental health apps and internet resources, ranging from meditation and mindfulness apps to symptom diaries and self-management tools such as cognitive behavioral therapy (CBT) [11]. While there is evidence for the efficacy of internet-based self-directed CBT and behavioral activation, evidence for many apps is limited, with inconsistent documentation of how apps are informed by evidence, as well as how they are accessible and tailored for underresourced groups [11-13]. Commentaries on next directions emphasize reframing tools as service enhancements and designing tools with end-user input, attending to the community and system contexts [14]. Some studies used consumer advisor input to tailor apps, such as sexual minority men to manage generalized anxiety and major depressive disorder [15] or consumer advisors’ partnered digital resources for youth or underresourced communities, but with limited evidence on impacts [16,17]. A recent review suggested greater uptake and effectiveness of digital resources with human contact in some studies, including a few rigorous randomized trials, although some showed no significant added effects [18]. Commentaries emphasize “solution-focused” approaches to digital mental health, with attention to user experience and an intervention target of engagement (usefulness, usability, satisfaction, use, contacts with others), consistent with community-engaged approaches [19-21].

### Digital Mental Health Tools and Together for Wellness Development

Given a history of partnered interventions, including those delivered during disasters [22,23], California Health Care Services Division of Behavioral Health included in its Federal Emergency Management Agency (FEMA)/Substance Abuse and Mental Health Services Administration (SAMHSA) a COVID-19 crisis counseling program, supporting free digital mental health resources (Together for Wellness/Juntos por Nuestro Bienestar [T4W/Juntos]) with partner input. T4W/Juntos features evidence-informed or evidence-based resources reviewed with lead agencies (Latinx, Black, Asian American, LGBTQ+, parent support for youths, older adults, persons with mental illness). The public website was developed iteratively, initially focusing on mindfulness, and adding resources vetted by community partners on coping with stress; grief; connecting to others; social justice issues such as racial discrimination; information on COVID-19; and resources for families with children, teachers, and older adults. Given the input, toolkits included visuals and videos, resources in multiple languages, and video orientations by provider and community partners; the initial partnered development process was previously described in a commentary [24].

This article describes further development of T4W/Juntos and results of a partnered evaluation, informed by principles of community-partnered participatory research (CPPR), including trust, respect, and two-way input [21]. The website and evaluation are informed by the technology acceptance model (TAM) and Behavioral Model for Vulnerable Populations, with COVID-19–related behavior changes [25-27]. The TAM explains use of technologies by professionals and patients, emphasizing reported engagement/satisfaction and comfort in use, contributing to actual use [25]. The Vulnerable Populations model emphasizes factors affecting underresourced groups [26], coupled here with COVID-19–related changes [27], to inform mechanisms of action (engagement, comfort in use) for promoting mental well-being, attending to the individual and system contexts [19,20].

The research questions for this study were as follows:

(1) What are specific individual and social factors that promote engagement and use of the T4W/Juntos website?

(2) Does reported engagement in and use of T4W/Juntos reduce symptoms of depression and anxiety (primary), and use of crisis hotlines and behavioral health services (secondary)?

Based on input from community and policy partners, we hypothesized that individuals with higher need (greater depression, anxiety symptoms) and more COVID-19–related behavior changes (such as social distancing) and COVID-19–related stressors (such as loss or financial stress) would engage more in website use, and that engagement and use between baseline and follow-up would be associated with reduced depression, anxiety, and use of hotlines at follow-up, as evaluated by participant surveys.

## Methods

### T4W/Juntos Resources

The website was iteratively reviewed by community partners in group meetings (largely by Zoom) in English and Spanish. Meetings included representatives of 11 agencies, youth and older adult advisory groups, and involved interaction with investigators and technology design leaders. Resources were reviewed and updated. Suggestions led to modifications in design, addition of other languages, and others. Based on this feedback, 133 resources were selected for T4W/Juntos: websites (n=62), videos (n=18), YouTube links (n=2), PDFs (n=32**)**, apps (n=10), and hotlines (n=9). The 6 main categories with examples are illustrated in Table 1. The website is available in 13 languages (videos in Mixteco were published in the summer of 2022). Once resources were made available on the website, we used an iterative process through focus group sessions (N=4) that consisted of youth (n=8) and older adult (n=10) advisors. Community partners were asked to provide feedback about their experience using the website and their review of resources. Each focus group was led by a moderator and notes were taken by members of the T4W/Juntos team. Feedback was communicated to the technology team and the website was revised/updated accordingly.

**Table 1:** Examples of digital mental health resources selected for the Together for Wellness/Juntos por Nuestro Bienestar website.

Resource			Description
**Learn About COVID-19**			
	Multilingual Resource Hub: TranslateCOVID.org^a^	COVID-19 information in over 40 languages on the disease, impacts, vaccines, tips to prevent the spread, and more. Created by the UCLA^b^ Asian American Studies Center	
	COVID-19 Guide for Trans people	COVID-19 guide for the transgender community and their families. Developed by the National Center for Transgender Equality	
	Covid-19 in Pregnancy^c^	Video featuring Yalda Afshar, MD, PhD, and Rashmi R Rao, MD, at UCLA, who explain COVID-19 and answer questions regarding pregnancy during this pandemic. Video created by UCLA Health	
	Covid Coach^d^	App created for everyone, including veterans and service members, to support self-care and overall mental health during the COVID-19 pandemic. Developed by the mobile mental health team of the National Center for PTSD^e^, Dissemination & Training Division	
	Be Safe and Healthy with Potter the Otter	A kid-friendly guide on staying safe during the COVID-19 pandemic starring First 5’s Potter the Otter. Created by First 5 LA	
**Soothe Anxiety and Stress**			
	Deep Breathing for Beginners^d^	YouTube video demonstrating deep breathing, and giving tips for improving oxygen uptake and reducing stress and fatigue. YouTube channel led by Michelle Kenway	
	UCLA Mindfulness App^d^	App on mindfulness in meditations that help reduce stress and anxieties, created by the UCLA Mindful Awareness Research Center	
	Mindshift App^a,d^	App based on cognitive behavioral therapy, developed to help you learn to relax and be mindful, develop more effective ways of thinking, and use active steps to take charge of your anxiety. Developed by Anxiety Canada	
	The Safe Place^d^	Minority mental health app geared toward the Black community, with meditations, breathing and exercise tips, coping with police brutality, and more. Developed by Jasmin Pierre	
	Stress Relief for Caregivers and Kids during COVID-19^d^	Guide with tips for parents and caregivers on how to take care of themselves and their families. Developed by the California Surgeon General’s office	
**Support Resilience**			
	123 Sesame Street: Resilience^d^	Webpage with videos, articles, and other resources for parents and caregivers, with tips for helping children manage anxiety during this time. Produced by Sesame Workshop	
	First Aid for Feelings^d^	A book of activities for children to understand the changes that are occurring in their lives due to COVID-19. The activities will provide emotional support and resilience skills. Developed by Denise Daniels, RN, MS; Scholastic; and the Yale Child Study Center	
	Dav Pilkey at Home	Webpage that offers children activities for reading, drawing, creating, as well as videos, featuring well-known cartoon characters. Developed by Scholastic	
**Cope with a Recent Loss**			
	Apart of Me^c^	Game designed by grief experts to help users come to terms with the loss they have suffered. Developed by Apart of Me	
	Healing After Death	App with guided meditations designed to assist you in supporting the spirit of someone you love who has passed away. Developed by Vanessa Callison-Burch	
	VA: Deal with Loss^c,d^	Guide for taking care of yourself after a loss. Developed by the National Center for PTSD	
**Connect with People & Support Social Justice**			
	TEACH. PLAY. LOVE. - Important Conversations About Social Justice for Kids^c,d^	Podcast led by child development experts who share strategies to help you teach your child about diversity, equity, and inclusion. Developed by Bright Horizons	
	Covid-19 Resources to Stand Against Racism	Webpage with a list of resources and helpful tips, primarily for AAPI^f^ communities, in light of the increased hate crimes that occurred during the pandemic. Developed by Asian Americans Advancing Justice	
	Explore Justice	A video series designed to help us unpack and examine the current and historical perspectives that shape social justice. Developed by 211LA	

^a^Evidence-based.

^b^UCLA: University of California, Los Angeles.

^c^Expert opinion.

^d^Evidence-informed.

^e^PTSD: posttraumatic stress disorder.

^f^AAPI: Asian American and Pacific Islander.

### Evidence Basis

As shown in Table 1, most resources are evidence-informed or evidence-based for specific populations, rather than for public prevention. For example, there are studies of effectiveness of mindfulness on attentional and emotional self-regulation [28]; one study of community health promotion used a pre-post design [29] and another was a randomized trial for depressed English and Spanish adults, which was found to reduce depression [28]. Mindfulness is thus considered to be “evidence-informed” for public prevention. Some resources are based on CBT and mindfulness (such as Mindshift App and Sanvello), whereas others provide information (eg, on depression) with resources for subgroups (eg, children).

### Design

The website evaluation was conducted in collaboration with 11 California community-based agencies representing diverse populations. Each agency gave input in planning meetings on survey measures and design, signed an agreement, and received US $1000 for their work to invite participants for the survey, including clients, partners, and staff, as groups impacted by COVID-19. For agencies with large lists of potential participants, random selection tools were provided. Assuming a 50% response rate, we encouraged the agencies to invite 80-100 individuals to ultimately enroll 30-40 per agency; that number was later expanded to 70 per agency to increase enrollment. The goal was to have 300-350 participants review the website and complete the baseline survey online; of these, 10% would be invited for a qualitative telephone interview 2 weeks later and all would be invited for a 4-6–week online follow-up survey. The goal was to apply the TAM/Vulnerable Population Model to describe engagement in website use [30], and predictors of engagement and anxiety/depression (Generalized Anxiety Disorder scale-2 [GAD2]/Patent Health Questionnaire-2 [PHQ2]), including demographics, services use, and COVID-19–related behavior changes and stressors [22,27,31-33]. For the evaluation, we created a duplicate version of the entire public-access website that allowed us to track enrollment by the inviting agency. Potential participants were emailed invitations (with 1-2 reminders) and a link to the eligibility screener (participants needed to have online access, speak English or Spanish, be aged 18+ years). Eligible participants who consented online were registered, given a link to the duplicate website that they were asked to visit, and asked to complete a baseline survey after visiting the website. Those who completed the baseline survey and consented to follow-up were invited to participate online surveys 4-6 weeks later, and (if selected) to be contacted for a qualitative interview 2 weeks after baseline (10% of total sample). Surveys were provided in English or Spanish. The survey period was September 20, 2021, to April 4, 2022, for the baseline, and October 22, 2021, to May 17, 2022, for follow-up.

### Ethics Approval

The study was reviewed and approved by the University of California, Los Angeles Institutional Review Board for Human Subjects (20-002163-AM-00008). All participants provided informed consent online and were advised that participation was voluntary, data would be deidentified, their contact information would be used for follow-up, and linking data (an ID code) would be stored on a secure server separately from study data. Informed consent was affirmed at follow-up assessments. For each participation event (survey, interview), participants received a US $25 electronic gift card. Inviting agencies were not informed of the participation of individuals. The study team used the separately stored contact information to invite participants to follow-up. Participation in each activity was voluntary.

### Measures

#### Baseline

Demographics included age in years, gender (female, male, genderqueer, questioning, trans man, trans woman, other/unknown, not stated), sexual orientation (gay/lesbian, bisexual/pansexual, queer, not sexual/none, questioning, other/unknown, not stated), race/ethnicity (American Indian/Native American/Alaskan; Black/African American/African; East Asian; South East Asian; Hispanic, Latino/Spanish Origin; Middle Eastern; Pacific Islander; white/Caucasian; unknown/not stated), website language preference (English, Spanish, Cantonese, Vietnamese, Tagalog, Mandarin, Korean, Japanese, Russian, Farsi, Armenian, Arabic, Mixteco, other/unknown/not stated), education (some high school or less, high school graduate/equivalent, vocational/certificate, some college, college graduate, graduate school), and zip code [22,34-36]. Two mental health stigma items were also included at baseline (scored on 5-point Likert agreement scales plus “don’t know”) [22].

#### Baseline and Follow-up

Depression and anxiety screeners included responses to the PHQ2 and GAD2 (range 0-6; score≥3 considered moderate depression/anxiety) [32,33]. Services use was assessed for the prior 6 months in the baseline survey and for the prior 1 month in the follow-up survey, including any hotline use for behavioral health (any and number of calls) and any behavioral health services (emergency room, primary care, mental health, substance use visits; hospitalization, residential care) [22]. Pandemic-related stress at baseline was assessed according to an adapted version of the COVID-19 Stress Scale [37], including COVID-19–related behavior changes (nine items: no changes, social distancing, isolation, caring for someone at home, working from home, not working, change in health care services, following media coverage on COVID-19, and changing travel plans). The overall impact of the pandemic was assessed over five categories (plus decline to answer). COVID-19 stressors [19] included having COVID-19; fear of acquiring or spreading COVID-19; worrying about others; stigma or discrimination; financial, food, and home insecurity; frustration, depression, anxiety, alcohol, sleep, and sexual activity change; confusion about COVID-19; other difficulties (not contributing to greater good, having social/emotional support, having financial support from others). Additional items included were vaccination status (yes/no), vaccine acceptability (5-point Likert agreement plus don’t know), date of survey completion, and racial/ethnic discrimination during the COVID-19 pandemic (assessed at baseline only: 6 items, 4-point Likert scale) [25,35].

The use of and engagement in the website at baseline were assessed according to website satisfaction, relevance of content, and ease of use, along with comfort in using the website (each with a 5-point Likert agreement scale plus “don’t know”). At follow-up, use and engagement were assessed according to actual website use in the prior month, including any use (yes/no); frequency/download and use of any of the six main categories, each with five response options (did not use, used some, used and valuable, used and very valuable, used and would recommend to anyone); and if recommended website to others (yes/no), number of times and which resource categories were recommended [22,25,31].

### Statistical Analyses

We performed descriptive analyses to describe the sample using means (SDs) for continuous variables, and counts and percentages for categorical variables. To describe predictors of engagement/use, we fit linear regression models for continuous variables (eg, mean engagement score) and logistic regression models for dichotomous variables (eg, used website). For preliminary bivariate analyses, predictors included demographics, need, stigma, COVID-19–related behaviors and stressors, and behavioral health services use (including predictors of follow-up nonresponse). The final regression model included predictors that were significant in preliminary models at *P*<.05.

For website use in the month before follow-up, we used logistic regression for any use and linear regression for total score of use across the six main categories. We examined predictors at follow-up of having PHQ2≥3 and GAD2≥3, and as sensitivity analyses, the post minus pre difference in PHQ2 and GAD2 scores. For secondary analyses, we examined predictors of hotline use and any behavioral health services use (outpatient, inpatient, rehabilitation, hotlines) in the month before follow-up; an imputed version of inpatient/rehabilitation was used for sensitivity analysis, assigning 0 if the items were skipped but other use items answered. We initially examined all measures as predictors in bivariate analyses, including predictors of follow-up nonresponse. We then fit regression models with significant predictors to inform final models. For final follow-up models, we conducted sensitivity analyses using inverse propensity weighting of data for predictors of nonresponse at baseline (age) and for follow-up after baseline [38,39].

## Results

### Participant Characteristics

By May 31, 2022, 495 individuals had completed the eligibility screener. Of the 446 that were eligible, 367 (82.3%, 6-69 per agency) consented, with no significant difference in mean age (**P*=*.56) between those that did not consent (38.7, SD 12.8 years) and did consent (39.7, SD 13.93 years). Of the 367 who consented, 315 (85.8%) completed baseline surveys, with a significantly lower mean age (*P*=.002) for completers (38.8, SD 13.5 years) than noncompleters (45.3, SD 15.1 years).

Of the baseline participants, 72.6% were female, 21.3% self-reported as LGBTQ+, 44.0% were Hispanic, 17.8% African American, 26.9% non-Hispanic white, and 11.4% endorsed another ethnicity. The sample’s mean age was 38.8 years with 110 (34.9%) aged 18-30 years and 18.5% had a high school education or less. Of the baseline participants, 32.7% screened positive for moderate anxiety or depression (GAD2/PHQ2≥3) and 61.2% had used behavioral health services in the prior 6 months. For COVID-19–related behavior changes, the mean number selected of the 9 listed was 3.9 (SD 2.0). For COVID-19 stressors, the mean selected of the 19 listed was 6.8 (SD 4.1) (Table 2).

**Table 2:** Characteristics at baseline, overall, and by follow-up status.

Characteristics		Baseline (n=315)	Had follow-up (n=193)	No follow-up (n=122)	*t* or *χ*^2^ (*df*)^a^		*P* value	
Age (years), n responses, mean (SD)		315, 38.8 (13.5)	193, 38.1 (13.0)	122, 39.9 (14.3)	–1.18 (313)		.24	
**Gender, n (%)**						10.13 (2)		.006
	Responses, n	310	189	121				
	Female	225 (72.6)	130 (68.8)	95 (78.5)				
	Male	70 (22.6)	53 (28.0)	17 (14.1)				
	Other	15 (4.8)	6 (3.2)	9 (7.4)				
**Sex minority, n (%)**						0.51 (1)		.48
	Responses, n	300	185	115				
	Yes	64 (21.3)	37 (20.0)	27 (23.5)				
	No	236 (78.7)	148 (80.0)	88 (76.5)				
**Race, n (%)**						13.04 (3)		.005
	Responses, n	298	184	114				
	Hispanic	131 (44.0)	68 (37.0)	63 (55.3)				
	Black/African American	53 (17.8)	32 (17.4)	21 (18.4)				
	White/Caucasian	80 (26.8)	61 (33.2)	19 (16.7)				
	Other	34 (11.4)	23 (12.5)	11 (9.6)				
**Education, n (%)**						5.15 (4)		.27
	Responses, n	313	192	121				
	Less than high school	23 (7.3)	10 (5.2)	13 (10.7)				
	High school graduate	35 (11.2)	21 (10.9)	14 (11.6)				
	Some college	87 (27.8)	56 (29.2)	31 (25.6)				
	College	119 (38.0)	78 (40.6)	41 (33.9)				
	Graduate school	49 (15.7)	27 (14.1)	22 (18.2)				
**Language prefer to use on the website, n (%)**						0.02 (2)		.99
	Responses, n	313	191	122				
	English	260 (83.1)	159 (83.2)	101 (82.8)				
	Spanish	40 (12.8)	24 (12.6)	16 (13.1)				
	Other	13 (4.2)	8 (4.2)	5 (4.1)				
**PHQ2^b^ or GAD2^c^≥3, n (%)**						0.92 (1)		.34
	Responses, n	312	190	122				
	Yes	102 (32.7)	66 (34.7)	36 (29.5)				
	No	210 (67.3)	124 (65.3)	86 (70.5)				
PHQ2 score, N responses, mean (SD)		311, 1.6(1.5)	190, 1.7(1.4)	121, 1.4(1.5)	1.87 (309)		.06	
**PHQ2≥3, n (%)**						2.82 (1)		.09
	Responses, n	311	190	121				
	Yes	61 (19.6)	43 (22.6)	18 (14.9)				
	No	250 (80.4)	147 (77.4)	103 (85.1)				
GAD2 score, N responses, mean (SD)		312, 1.8(1.7)	190, 1.9(1.7)	122, 1.6 (1.5)	1.81 (310)		.07	
**GAD2≥3, n (%)**						0.37 (1)		.54
	Responses, n	312	190	122				
	Yes	80 (25.6)	51 (26.8)	29 (23.8)				
	No	232 (74.4)	139 (73.2)	93 (76.2)				
Stigma score, N responses, mean (SD)		290, 2.8 (1.0)	181, 2.9 (1.0)	109, 2.7 (1.0)	0.96 (288)		.34	
Engagement score (3 items)^d^, N responses, mean (SD)		304, 4.0 (0.7)	188, 4.0 (0.8)	116, 4.1 (0.7)	–2.03 (302)		.04	
**Do not feel comfortable using this website, n (%)**						1.14 (4)		.89
	Responses, n	305	188	117				
	Strongly disagree	106 (34.8)	66 (35.1)	40 (34.2)				
	Disagree	108 (35.4)	65 (34.6)	43 (36.8)				
	Neither agree nor disagree	22 (7.2)	12 (6.4)	10 (8.5)				
	Agree	37 (12.1)	25 (13.3)	12 (10.3)				
	Strongly agree	32 (10.5)	20 (10.6)	12 (10.3)				
**Any service use for emotional, mental health, alcohol, or drug problems, n (%)**						0.44 (1)		.51
	Responses, n	197	130	67				
	Yes	121 (61.4)	82 (63.1)	39 (58.2)				
	No	76 (38.6)	48 (36.9)	28 (41.8)				
**Any service use for emotional, mental health, alcohol, or drug problems, imputed, n (%)**						2.07 (1)		.15
	Responses, n	240	152	88				
	Yes	121 (50.4)	82 (53.9)	39 (44.3)				
	No	119 (49.6)	70 (46.1)	49 (55.6)				
Total number of COVID-19–related changes, N responses, mean (SD)		315, 3.9 (2.0)	193, 4.0 (1.9)	122, 3.6 (2.1)	1.75 (313)		.08	
Total number of COVID-19 stressors experienced, N responses, mean (SD)		315, 6.8 (4.1)	193, 7.3 (4.3)	122, 6.0 (3.8)	2.81 (313)		.005	

^a^*χ*^2^ tests were used for categorical variables and *t* tests were used for continuous variables to compare groups with and without a follow-up response.

^b^PHQ2: Patient Health Questionnaire-2 item.

^c^GAD2: Generalized Anxiety Disorder-2 item scale.

^d^Items (ease of use, relevance of topics, satisfaction) were averaged as mean engagement based on 5-point Likert scales.

### Website Engagement

Of all participants, 87.1% agreed the website was easy to use, 80.5% found the topics to be relevant, 85.9% were satisfied with the website, and 70.2% were comfortable using the website. Three items (ease, relevance, satisfaction) were averaged to obtain the mean engagement score with standard Cronbach α=.736; comfort using the website was considered a separate measure. In final regression analyses, predictors of higher baseline engagement included Hispanic versus other race/ethnicity (*β*=.27, 95% CI .10-.44; *P*=.002) and COVID-19–related behavior changes (*β*=.09, 95% CI .05-.13; *P*<.001). At baseline, 83.1% (260/313) of participants reported English as their preferred language for website use versus 12.8% (40/313) Spanish or 4.2% other (13/313) (Table 2). Predictors for comfort using the website in final regression analyses were preferring English compared to other languages (odds ratio [OR] 5.57, 95% CI 2.22-13.96; *P*<.001) and number of COVID-19–related behavior changes (OR 1.37, 95% CI 1.12-1.66; *P*=.002). In addition, those having overnight treatment for behavioral health within 6 months before baseline (n=8 inpatients, n=10 rehabilitation patients) had less comfort using the website (OR 0.15, 95% CI 0.03-0.69; *P*=.02).

### Follow-up Response and Predictors

Of the 315 participants completing the baseline survey, 193 (61.3%) completed follow-up surveys. Table 2 shows baseline factors in bivariate analyses that predicted follow-up survey completion (significance of differences between groups with and without follow-up response were evaluated with *χ*^2^ tests for categorical variables and with *t* tests for continuous variables). In final logistic regression models, baseline predictors of follow-up survey response were: (1) non-Hispanic white versus other race/ethnicity (OR 2.04, 95% CI 1.11-3.76; *P*=.02), (2) lower mean website engagement (OR 0.66, 95% CI 0.45-0.95; *P*=.03), and (3) greater number of COVID-19–related stressors (OR 1.07, 95% CI 1.01-1.14; *P*=.03). These variables were included as predictors in follow-up models and were excluded if not significant.

### Follow-up Website Use

Among the follow-up participants, 119/193 (61.7%) visited the website or used resources in the month prior to follow-up surveys. By category, the rate of resource use was 50.0% (96/192) for (1) Learn about COVID, 53.9% (103/191) for (2) Soothe Anxiety and Stress, 40.0% (76/190) for (3) Support Resilience in Kids and Families, 35.6% (68/191) for (4) Cope with a Recent Loss, 42.2% (81/192) for (5) Build Community and Connect with People, and 31.3% (60/192) for (6) Need to Talk to Someone? (Helplines). For any use in the month prior to follow-up, unique baseline predictors in the regressions were: PHQ2 score (higher=more use), called hotline for behavioral health before baseline (higher=more use), and greater alcohol use during COVID-19; these predictors were also significant when weighted for nonresponse (Table 3). The mean score of category use (1-5, from "no use" to "recommend to everyone") varied from a high of 2.30 (SD 1.45) for Soothe Anxiety and Stress to a low of 1.75 (SD 1.26) for Need to Talk to Someone; mean scores were significantly higher for Categories 1-3 and 5 than for 6; categories 1 and 2 than for 4 and 5; and category 2 than category 3 (all *P*≤.002). Predictors of higher total use summed across the six categories were: Hispanic ethnicity, baseline PHQ2 or GAD2 score ≥3, and caring for someone at home during COVID-19 (Table 3). COVID-19–related change in sexual activity was associated with reduced total use; these predictors were also significant with nonresponse weighting (Table 3).

**Table 3:** Final models for website use in the month before follow-up.

Variables		Main analysis (unweighted)			Sensitivity analysis (IPW^a^, nonresponse)		
		Statistic^b^	95% CI	*P* value	Statistic	95% CI	*P* value
**Any website use^c^**							
	Female	0.52	0.25 to 1.08	.08	0.61	0.27 to 1.35	.22
	PHQ2^d^>3	1.33	1.04 to 1.69	.02	1.44	1.12 to 1.85	.004
	Called hotline for behavioral health before baseline	6.50	1.68 to 25.24	.007	6.48	1.25 to 33.58	.03
	COVID-19 stressor, increased alcohol/substance use	0.26	0.11 to 0.60	.002	0.21	0.09 to 0.52	<.001
**Use of 6 categories^e,f^**							
	Hispanic or Latino (vs other race/ethnicity)	2.74	0.75 to 4.73	.007	2.56	0.41 to 4.72	.02
	PHQ-2 or GAD-2^g^≥3	3.39	1.39 to 5.40	.001	4.51	2.18 to 6.83	<.001
	COVID-related change, caring for someone at home	2.86	0.77 to 4.95	.008	3.24	0.97 to 5.51	.006
	COVID stressor, change in sexual activity	–3.58	–5.88 to –1.28	.002	–3.97	–6.04 to –1.91	<.001

^a^IPW: inverse probability weighting for nonresponse predictors at baseline and follow-up.

^b^The effect is presented as the odds ratio for any website use and as *β* for use of 6 categories.

^c^Analytical N=186 for main analysis, N=181 for sensitivity analysis.

^d^PHQ2: Patient Health Questionnaire-2 item.

^e^Analytical N=175 for main analysis, N=170 for sensitivity analysis.

^f^Total score across 6 categories (Learn about COVID, Soothe Anxiety and Stress, Supporting Resilience in Kids and Families, Cope with a Recent Loss, Build Community and Connect With People, and Need to Talk to Someone) each of which has 5 responses (not use to recommend to anyone).

^g^GAD2: Generalized Anxiety Disorder scale-2 item.

### Associations With Primary and Secondary Follow-up Outcomes

The main predictor of follow-up depression (PHQ2≥3) was baseline depression (Table 4). In addition, greater reported engagement in T4W/Juntos was associated with a lower likelihood of depression at follow-up (Table 4). PHQ2 scores can range from 0 to 6; for our sample, the baseline mean PHQ2 score was 1.6 (SD 1.5). Predictors of a post-pre change in the PHQ2 score included: (1) Caucasian/white versus other race/ethnicity, which was associated with an increase in PHQ2; (2) using the website resources in the month before follow-up, which was associated with a reduced post-pre PHQ2 score; and (3) having more COVID-19–related stressors at baseline, which was associated with a reduced post-pre PHQ2 score (Table 4). The findings were similar when weighted for nonresponse. For GAD2≥3 at follow-up, the main predictor was baseline GAD2. For a post-pre change in the GAD2 score, the main predictors were non-Hispanic white versus other race/ethnicity, age, and COVID-19–related stressors; however, only COVID-19–related stressors and age were significant in the analysis weighting for nonresponse (Table 4).

Predictors of using hotlines for behavioral health in the month before follow-up included: baseline depression or anxiety (PHQ2 or GAD2≥3) (*P*=.006) and use of hotlines prior to baseline assessment (*P*=.002). Greater baseline mean website engagement, greater comfort using the website at baseline, and greater mean total website use in the month before follow-up each predicted reduced hotline use at follow-up, which remained significant with nonresponse weighting (Table 5). For use of any behavioral health services (outpatient, inpatient, rehabilitation, hotlines) prior to follow-up, the main predictor in logistic regression was use of such services prior to baseline. Increased reported mean engagement with the website at baseline was associated with a borderline trend toward reduced probability of behavioral services use, which was significant when weighting for nonresponse (Table 5).

**Table 4:** Final models for follow-up Patient Health Questionnaire (PHQ2) and Generalized Anxiety Disorder (GAD2) associated with website use.

Variables		Main analysis (unweighted)				Sensitivity analysis (IPW^a^, nonresponse)		
		Statistic^b^	95% CI	*P* value	Statistic		95% CI	*P* value
**Follow-up PHQ2≥3^c^**								
	Engagement mean score, 3 items^d^ (baseline)	0.54	0.34 to 0.86	.01	0.55		0.33 to 0.91	.02
	PHQ2≥3 (baseline)	6.34	2.77 to 14.09	.04	6.53		2.78 to 15.84	<.001
**Follow-up GAD2^e^≥3**								
	GAD2≥3 (baseline)	13.65	6.06 to 30.72	<.001	11.45		4.81 to 22.27	<.001
**PHQ2 mean score post-pre change^f^**								
	Non-Hispanic white (vs other race/ethnicity)	.46	.03 to .90	.04	.44		.00 to .87	.049
	Visited T4W/Juntos^g^ or used resources month before follow-up	–.62	–1.04 to –.20	.004	–.52		–.93 to –.19	.02
	Total number of COVID-19 stressors (baseline)	–.07	–.12 to –.02	.004	–.08		–.13 to –.02	.005
**GAD2 mean score post-pre change^h^**								
	Non-Hispanic white (vs other race/ethnicity)	.44	.00 to .87	.048	.30		–.14 to .74	.19
	Age	.02	.00 to .03	.02	.02		.00 to .03	.049
	Total number of COVID-19 stressors (baseline)	–.06	–.11 to –.01	.02	–.08		–.13 to –.03	.002

^a^IPW: inverse probability weighting for nonresponse predictors at baseline and follow-up.

^b^The effect is presented as the odds ratio for follow-up scores ≥3 and as *β* for mean post-pre changes in scores.

^c^Analytical N=187 for main analysis, N=185 for sensitivity analysis.

^d^Items (ease of use, relevance of topics, satisfaction) were averaged as mean engagement based on 5-point Likert scales.

^e^Analytical N=187 for main analysis, N=187 for sensitivity analysis.

^f^Analytical N=181 for main analysis, N=176 for sensitivity analysis.

^g^T4W/Juntos: Together for Wellness/Juntos por Nuestro Bienestar.

^h^Analytical N=178 for main analysis, N=173 for sensitivity analysis.

**Table 5:** Final models for follow-up hotline and behavioral health service use.

Variables		Main analysis (unweighted)			Sensitivity analysis (IPW^a^, nonresponse)		
		OR^b^	95% CI	*P* value	OR	95% CI	*P* value
**Use of hotlines for behavioral health in month before follow-up^c^**							
	PHQ2^d^ or GAD2^e^≥3 (baseline)	7.89	1.79-34.77	.006	9.58	1.68-54.83	.01
	Using hotline for behavioral health prior to baseline	10.44	2.34-46.47	.002	10.75	1.98-58.38	.006
	Engagement mean score, 3 items^f^ (baseline)	0.36	0.17-0.76	.007	0.36	0.15-0.84	.02
	Comfort using website (baseline)	0.23	0.06-0.91	.04	0.18	0.04-0.91	.04
	Total score of website category use in past month before follow-up^g^	1.15	1.03-1.28	.01	1.15	1.07-1.23	<.001
**Use of any behavioral health services in past month before follow-up^h^**							
	Visited any behavioral health provider in 6 months prior to baseline	14.78	5.57-39.25	<.001	15.02	5.06-44.61	<.001
	Engagement mean score, 3 item (baseline)^f^	0.61	0.35-1.07	.08	0.59	0.35-1.00	.05

^a^IPW: inverse probability weighting for nonresponse predictors at baseline and follow-up.

^b^OR: odds ratio.

^c^Analytical N=174 for main analysis, N=172 for sensitivity analysis.

^d^PHQ2: Patient Health Questionnaire-2 item.

^e^GAD2: Generalized Anxiety Disorder-2 item.

^f^3 items (ease of use, relevance of topics, satisfaction) averaged as mean engagement; 5-point Likert scales for agreement.

^g^Total score across 6 categories (Learn about COVID, Soothe Anxiety and Stress, Supporting Resilience in Kids and Families, Cope with a Recent Loss, Build Community and Connect, and Need to Talk to Someone) each of which has 5 responses (not use to recommend to anyone).

^h^Analytical N=137 for main analysis, N=136 for sensitivity analysis.

## Discussion

### Principal Findings

This article presents an evaluation of engagement in and impact of free digital mental health resources developed with community advisor input to support well-being during COVID-19 in California. Informed by the TAM, Behavioral Health Model for Vulnerable Populations, COVID-19 stressors, and CPPR principles [21,25-27], we hypothesized that individuals with higher need (depression, anxiety) and more COVID-19–related behavior changes and stressors would engage more in website use, and that higher engagement and use would be associated with reduced depression, anxiety, and hotline use at follow-up. Findings on predictors of website use were somewhat consistent with these hypotheses, but the details differed for baseline and follow-up. For main outcomes, findings were consistent with hypotheses for depression and hotline use, in that greater engagement or use before follow-up was associated with lower follow-up depression and greater reduction in depression from baseline to follow-up, but not reduced anxiety, for the overall sample. The main findings were consistent in sensitivity analyses weighting for nonresponse, and a borderline significant trend for baseline website engagement was significantly associated with reduction in any behavioral health service use when weighting for nonresponse.

These findings suggest that recruiting participants through community agencies can generate a diverse sample in race/ethnicity, age, gender identity, and sexual orientation [1,6-8]. Baseline completers were somewhat younger than noncompleters, suggesting that more support for participation may be needed for older adults. Baseline website engagement was higher for those of Hispanic ethnicity, whereas comfort in website use was greater for people preferring English, suggesting that website modification or human support may be important for non-English speakers [18]. In addition, less comfort in use was associated with having a prior overnight behavioral health stay, which could reflect disability or need for support. Website use before follow-up was lower for those with greater alcohol use during the COVID-19 pandemic. Total website use across categories was higher for those caring for someone at home and was lower for those with a change in sexual activity during the pandemic. These findings highlight potential differences in website use patterns for specific COVID-19 changes, which is an area for future research. The main findings for depression and hotline use are largely consistent with policymaker goals for developing the website for prevention to reduce mental health needs and crisis calls.

### Limitations

Limitations included that, as a nonrandomized study, the findings could reflect selection effects or reverse causality, although the main finding of website use associated with reduced depression was robust in analyses of end status and post-pre change with and without nonresponse weighting. Higher use of this website could be associated with use of other websites that could have had an unmeasured (confounding) effect on outcomes. Further, the study used a convenience sample recruited by partnering agencies in one state representing diverse populations rather than a general population sample during a specific period of the pandemic (September 2021-May 2022). While effects of COVID-19 evolved over time, during this period, the date of survey completion was not significant and not included in final models. Future research may include experimental designs to clarify the causation and mechanisms of action. Further, the study had a moderate response at follow-up (61.3%). Predictors of response (non-Hispanic white, lower baseline engagement, greater number of COVID-19 stressors) were included as predictors in final models if significant, with weighting for nonresponse in sensitivity analyses. Recruited participants included clients, partners, and providers of partnering agencies, and we did not track participant roles; however, baseline education data suggest the sample included some nonproviders. In future research, it may be important to include demographic or cultural differences in stigma of mental health that may affect website use to inform the tailoring of resources, and it may be important to evaluate the added value of human support to enhance website use and survey response, particularly for non-English speakers or older adults [18].

### Public Health Implications

Given the consistency of main outcome findings with hypotheses and policy consumer advisor goals for developing digital resources, the findings were considered by policy partners as encouraging for further website development and evaluation as next steps, including expansion to youth/young adults. Descriptive findings reinforce the importance of community agency partnerships to achieve a diverse sample. These findings also raise the issue of potential impacts for diverse populations of prevention-oriented, free digital mental health resources during COVID-19 to reduce depression and crisis hotline use, an important issue for future randomized trials or quality improvement initiatives with public health implications. The consumer advisor engagement in this study reinforces the public health value of a partnered participatory effort in intervention development and evaluation. The findings suggest next steps for research in public health, such as exploring human support for digital resources, especially for non-English speakers, even for a website available in 13 languages. Although this represents only a preliminary evaluation, given limited data on digital mental health for preventive public health goals [10], the findings may inform the next steps for development of resources and evaluation efforts to inform public mental health prevention.

## Abbreviations

CBT: cognitive behavioral therapy
CPPR: community-partnered participatory research
FEMA: Federal Emergency Management Agency
GAD2: Generalized Anxiety Disorder-2 item scale.
LGBTQ+: lesbian, gay, bisexual, transgender, queer/questioning and others
OR: odds ratio
PHQ2: Patient Health Questionnaire-2 item
SAMHSA: Substance Abuse and Mental Health Services Administration (
T4W/Juntos: Together for Wellness/Juntos por Nuestro Bienestar
TAM: technology acceptance model
